# Strategies for surgical reconstruction of complex elbow deformities based on patient-specific instruments – a case series and narrative review

**DOI:** 10.1515/iss-2025-0024

**Published:** 2025-11-17

**Authors:** Sabine Manuela Irene Roth, Christian David Weber, Frank Hildebrand, Heide Delbrück

**Affiliations:** Department of Orthopaedics, Trauma and Reconstructive Surgery, University Hospital RWTH Aachen, Aachen, Germany

**Keywords:** patient-specific instruments, elbow, neglected monteggia, cubitus varus, multiple hereditary exostoses, 3D

## Abstract

**Objectives:**

The application of 3D-based methods for deformity correction of the upper extremity, including patient-specific instruments (PSIs), is attracting increasing attention in clinical practice and requires the collaboration of orthopaedic surgeons and medical engineers.

**Methods:**

The management of various elbow deformities using 3D-based surgical methods, including PSIs, is presented. A narrative literature review with a defined search strategy using PubMed as a database was conducted to identify further applications around the elbow that have been published to date.

**Results:**

This case series presents with one case each the correction of the following deformities: cubitus varus, dorsal radial head subluxation, translational deformity of the distal humerus, cubitus valgus (CVL) and radial head dislocation in hereditary multiple exostoses (HME). The Supplementary literature with 28 studies presents further deformities that were corrected on a 3D basis: intra-articular fracture of the capitulum radii, extension deformity of the distal humerus and malunited radial neck fractures. These 3D-based procedures have been used most frequently to correct cubitus varus and are superior to conventional methods in terms of accuracy in comparative studies.

**Conclusions:**

The application of 3D-based planning methods, including PSIs use, offers excellent opportunities and accuracy for corrective osteotomies around the elbow. In addition to the correction of cubitus varus, extension to the correction of chronic dislocation of the radial head after Monteggia fracture and HME appears promising. The future implementation of 3D-based planning based on magnetic resonance imaging (MRI) would be highly desirable.

## Introduction

The elbow joint is considered complex, where three joint partners with relatively small joint surfaces are exposed to large lever forces. Minimal deviations from the anatomical norm can lead to chronic pain and irreversible damage [1]. The use of 3D-supported planning and execution of corrective osteotomies in orthopaedics and trauma surgery is increasingly being integrated into everyday clinical practice, especially in the upper extremity, although it is not yet a standard procedure. In the upper extremity, the corresponding results have been published primarily for the outcome of corrections to the distal radius and forearm [2], [3], [4], [5]. When 3D-supported corrections in the elbow area are presented, they are primarily performed in the case of cubitus varus (CVR) deformities following supracondylar humerus fractures [6], 7]. However, radial head dislocation in the context of a neglected Monteggia fracture can also be considered a good indication, as up to 28 % of Monteggia fractures are missed [8]. Further indications involve a subluxation or dislocation of the radial head due to hereditary multiple exostoses (HME) (in 22–33 % of the cases) [9], [10], [11] as well as a cubitus valgus (CVL) after radial condyle fractures (in 4–8% of cases), often accompanied by ulnar nerve palsy in the further course [12], 13].

This study aimed to present the possibilities of complex corrections of the elbow region with 3D reconstruction and surgical simulation or further 3D-printed patient-specific instruments (PSIs). The method is based on interdisciplinary cooperation between orthopaedic surgeons and medical engineers. The current state of knowledge is presented based on our case series and a narrative literature review.

## Methods

### Case series

To illustrate the wide range of possible applications, five selected cases with representative clinical situations of complex bony elbow deformities treated by 3D-based reconstruction methods or PSIs were retrospectively presented as case descriptions with medical history, pre- and postoperative clinical and radiological situations, and explanations of surgery planning and implementation.

### Planning of surgery and guides in collaboration between orthopaedic surgeon and medical engineer

All planning procedures and surgeries were performed by the senior author in collaboration with the medical engineers of the company Materialise, Leuven, Belgium. Computed tomography (CT) scans of the entire affected humerus and forearm, as well as the opposite side, were obtained. The examination was carried out according to the specified scan protocol from Materialise, Leuven, Belgium (slice thickness humerus 1.25 mm, forearm 0.625 mm) [14]. Segmentation and 3D reconstruction were carried out by the company’s planning service. For an ideal reconstruction of the pathological side and as a reference, the healthy opposite side was mirrored; in the case of bilateral disease, an age-appropriate reference model was used. Via video conference with the medical engineers the surgeon determined the optimum osteotomy height, plate type and position so that the side to be operated on corresponded to the opposite side of the reference model. Planning and 3D printing of the PSIs were then carried out by Materialise. A comprehensive planning protocol was established for each step of the surgery and provided to the surgeon [15].

### Methods of narrative literature review

A narrative literature review was conducted to determine the current state of knowledge on the use of PSIs in the elbow region.

MEDLINE (PubMed) was used as the database. The search was performed on April 10th, 2025. The search terms are listed in Table 1.

**Table 1:** Search terms.

Health condition of interest respectively location	Procedure I	Procedure II
Malunion humerus Malunion forearm Upper extremity Elbow Neglected monteggia Missed monteggia Radial head luxation Radial head subluxation Radial head dislocation Cubitus varus Cubitus valgus Hereditary multiple exostoses	Patient-specific instruments Patient-specific implants Guides 3D	Osteotomy
All terms combined with ‘OR’	All terms combined with ‘OR’	
All results combined with ‘AND’		

Two reviewers (HD and SR) independently selected the articles for inclusion in the literature review. The inclusion criteria used for article selection were broadly defined according to the review objectives (Table 2). No case reports with objective data were excluded to provide a broad overview of the existing literature and the cases described in this field.

**Table 2:** Inclusion and exclusion criteria for title/abstract and full-text screening.

Inclusion criteria	Exclusion criteria
All publication types regarding – 3D-based surgical techniques on the humerus and forearm that affect: – Elbow joint position and function in particular: – Cubitus varus and valgus and radial head position	Arthroplasty Hand reconstruction Distal radius reconstruction Forearm osteotomies without effects on the elbow No English language Tumour surgery Other than journal article Distal radioulnar joint No 3D techniques Veterinary Cadaver No full text available Surgery on models [16]

## Case presentations

### Case 1: Radius head subluxation and cubitus varus after elbow fracture in childhood

A 38-year-old patient presented to our department with persistent pain in the right elbow. Furthermore, he reported that he had broken his arm as a child in Syria. No further information was available regarding this injury. The fracture was treated conservatively. Clinical examination revealed a free range of motion (ROM), posttraumatic cubitus varus and dorsoradial pain with a punctum maximum at the radial head.

**Figure 1:**
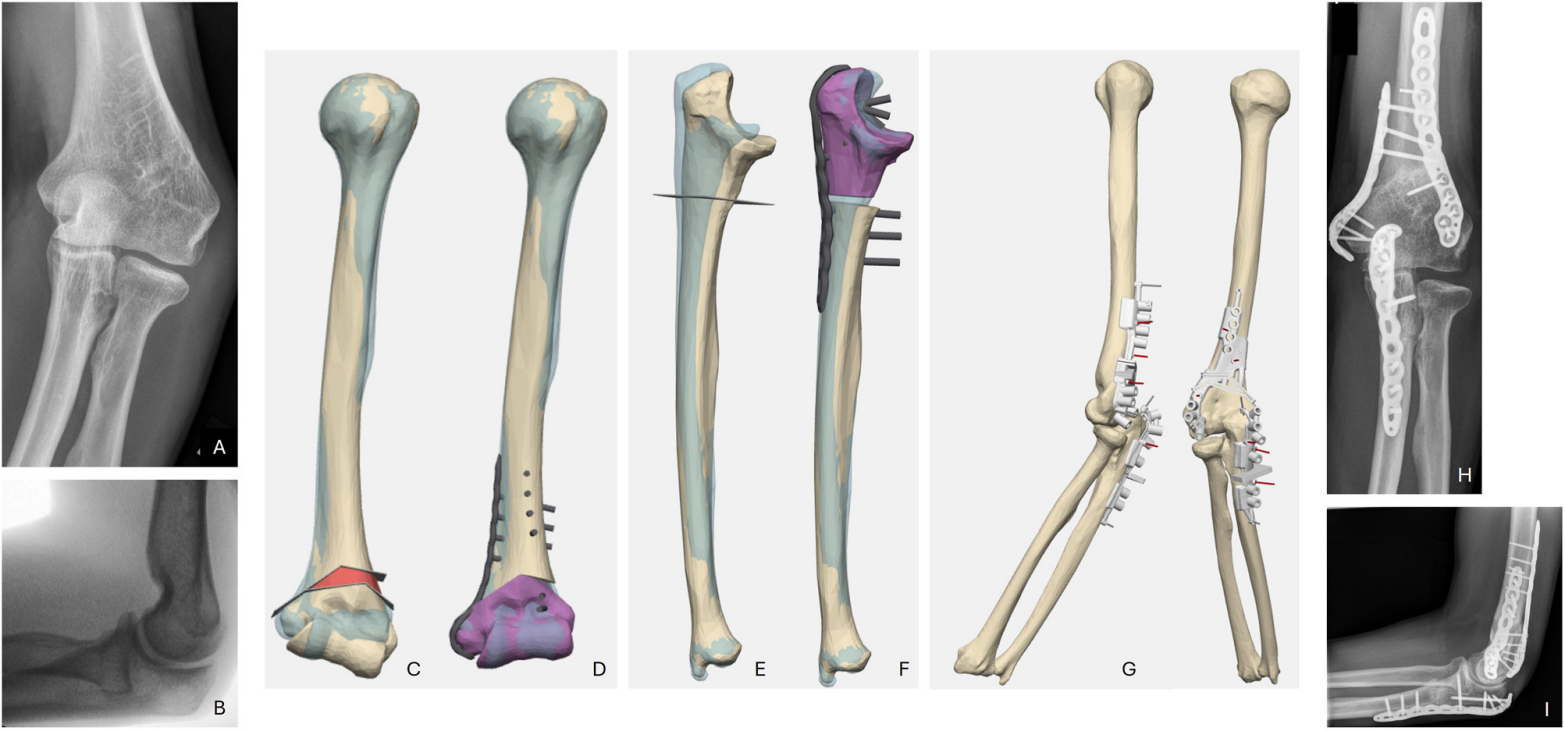
Case 1: A preoperative anterior-posterior radiograph revealed cubitus varus; B preoperative lateral radiograph revealed dorsal radial head subluxation; C 3D planning of supracondylar humerus osteotomy; D planned outcome of humerus osteotomy; E the ulnar overlay demonstrated the malposition (opposite side blue); F planned outcome of ulnar osteotomy; G lateral view with cutting and drilling guides revealed dorsal radial head subluxation; H healed osteotomies in ap radiograph; I healed osteotomies in lateral radiograph with good position of radial head.

The preoperative radiograph showed, in addition to the cubitus varus, dorsal subluxation of the radial head. A CT scan of both upper arms with subsequent 3D reconstruction and comparative analysis revealed varus malalignment of the distal humerus on the left by 30°, as well as an ulnar malalignment in the sense of slight retrocurvation and shortening. A corrective osteotomy was performed on the ulna, lengthening it and correcting its retroversion. In addition, corrective osteotomy of the distal humerus was carried out to correct the cubitus varus (Figure 1)

Postoperative CT revealed an exact match between 3D planning and surgical results. After one year and three months, material removal was performed. Six years after the surgery, the patient reported a very good clinical outcome, with no pain and full recovery. The patient exhibited a free ROM (Ex/Flex 0/0/140°, Pro/Sup 90/0/90).

### Case 2: Translation deformity after corrective osteotomy of a malunited supracondylar fracture

The patient had a supracondylar fracture at the age of six. The fracture was initially treated conservatively. At the age of 18 years, she experienced a posttraumatic elbow deformity with severe pain, which impaired her ability to lift loads. Therefore, she underwent two operative treatments in Madagascar. Due to persistent pain, inability to lift loads and persistent deformity, the patient presented to our hospital at the age of 27 years. Clinically, there was a ‘kink’ in the arm axis, which bothered the patient significantly. Therefore, she hid her arm under appropriate clothing. There was a slightly reduced ROM (Ex/Flex 0/5/110, Pro/Sup 90/0/90) and a very prominent medial plate.

**Figure 2:**
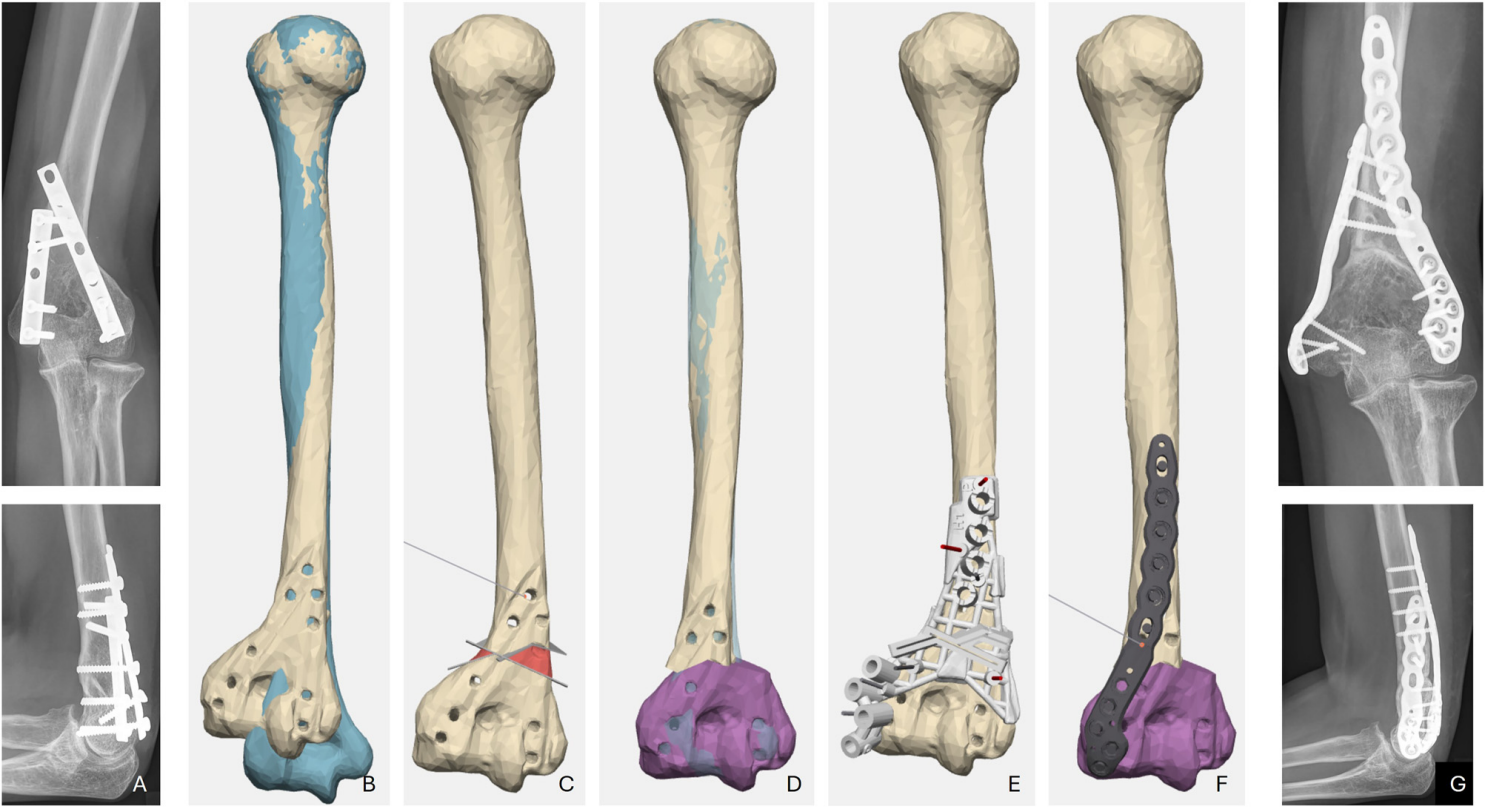
Case 2: 3D simulations view from dorsal; A preoperative radiography; B superimposition of healthy right side (blue) and deformed left side; C planned osteotomy; D planned outcome; E drilling and cutting guide; F planned plate position; G final position with healed osteotomy.

Preoperative radiography revealed radial translation malalignment. Multidimensional corrective osteotomy with 3D printed drilling, sawing and repositioning guides and double plate osteosynthesis was performed (Figure 2)

Four years after the surgery, the patient was delighted with the cosmetically straight arm, a good ROM with Ex/Flex 0/10/130 and free pronation and supination. She was pain-free and could put full weight on her arm.

### Case 3: Cubitus valgus cause of malunion of elbow fracture in early childhood

On initial presentation, a 33-year-old woman reported a fall in early childhood with a fracture of her right elbow, which was treated conservatively in her home country, Turkey. Over the years, an increasing deformity has developed. Furthermore, she experienced numbness in her little and ring fingers and a loss of strength in her forearm and hand. She had been unable to perform her job as a saleswoman for several months. Clinical examination revealed a CVL of 60°, a scar over the radial elbow joint, very good ROM with a slight extension deficit of 5° and unrestricted pronation and supination. No pain was observed during the movement test. Electroneurography revealed older damage to the ulnar nerve.

**Figure 3:**
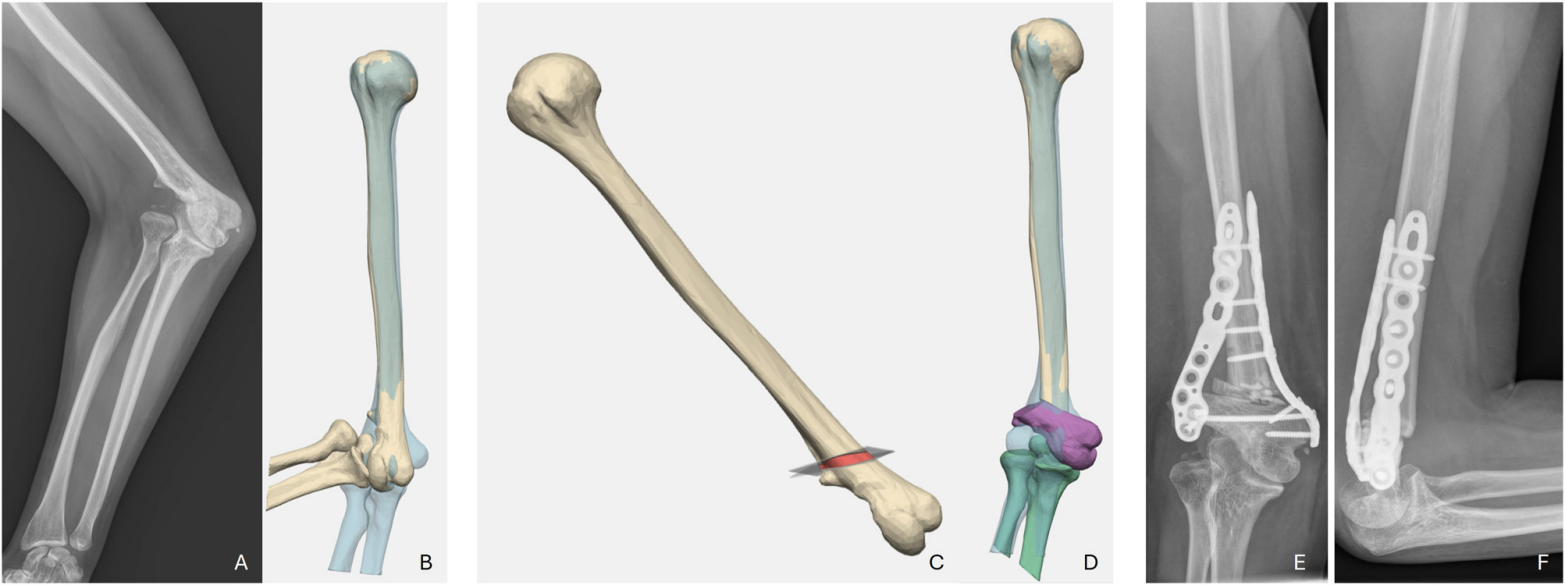
Case 3. A preoperative radiography strong anterior-posterior view shows 60° cubitus valgus and the capitulum radii is missed; B preoperative 3D reconstruction (blue opposite site); C simulated osteotomy with wedge; D simulated correction – the ulnar epicondylar pillar is rotated under the humeral shaft; E and F postoperative radiographs.

Radiography revealed a severe trochlea humeri deformity and a complete absence of capitulum radii. The 3D reconstruction was only used for planning purposes to gain an impression of the deformity and to play through possible correction options. Open or closed wedge osteotomies did not produce good correction results. Finally, the distal humeral fragment was rotated so that the ulnar epicondylar pillar became the contact partner for the proximal humeral shaft. Surgery with guides was not necessary in this case. Intraoperatively, a severely constricted ulnar nerve was observed, which was significantly thickened after release. Postoperative radiography showed good humeroulnar alignment (Figure 3)

At the follow-up examination, 8 weeks after the operation, the patient reported no further neurological restrictions. She was very satisfied with the surgery and demonstrated an unrestricted ROM.

### Case 4: Dorsal subluxation of the radial head most likely after neglected Monteggia fracture

The patient had sustained a forearm fracture as a child. At the age of 18 years, she noted a deficit in elbow flexion. An arthroscopy of the elbow was performed. It showed second-degree chondromalacia, and a free articular body was found in the radiohumeral joint and removed. She presented to our hospital at the age of 18 years with persistent pain in the elbow, which occurred particularly after driving a car. She also reported persistent elbow instability when propping up. Clinically, there was a good ROM with a ‘click’ in the humeroradial joint during extension and flexion, with a 10° restriction of flexion. The arm axis appeared in a slight varus, and there was a corresponding prominence in the region of the radial head.

**Figure 4:**
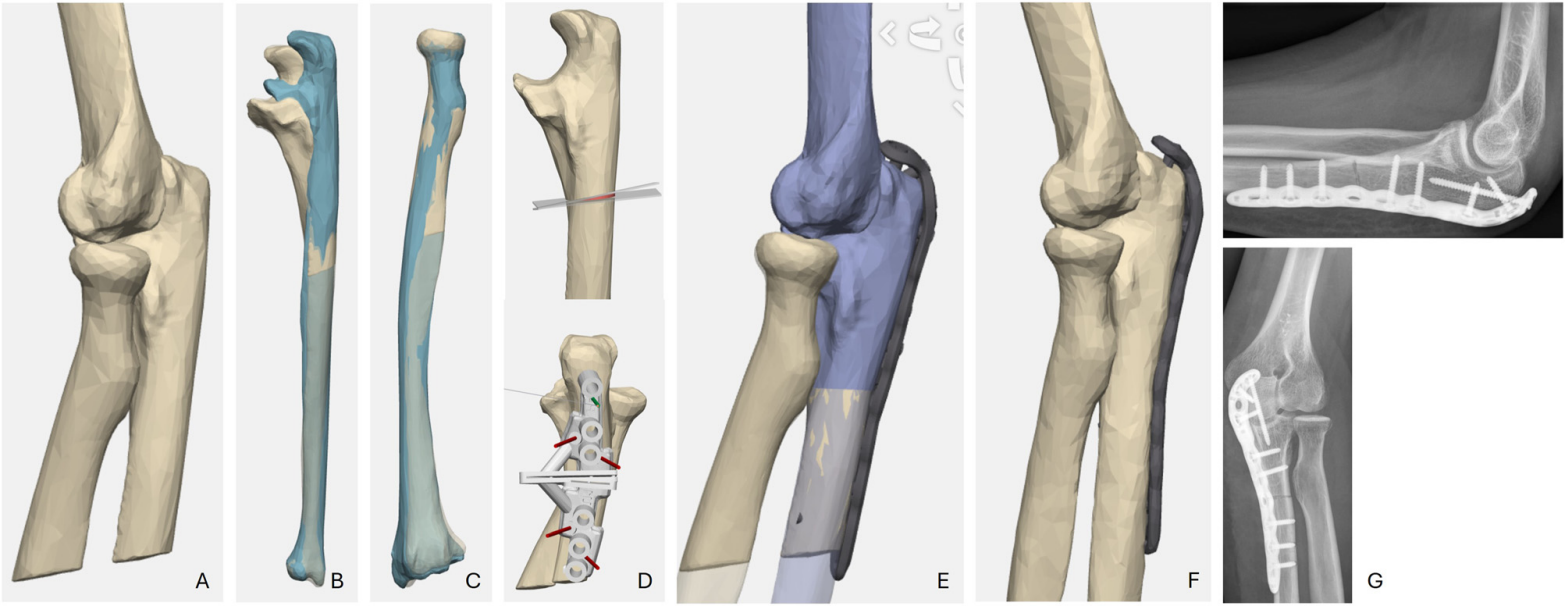
Case 4. A strong lateral view of preoperative CT revealed a malposition of radial head; B comparison of affected and non-affected ulna (healthy side blue); C radius comparison is equal-sided; D planned ulnar osteotomy and drilling and cutting guide; E planned radius head position strictly under the capitulum radii; F achieved position of radius head; G postoperative radiographs.

Based on the fracture history, we performed a CT scan of both forearms which demonstrated dorsal subluxation of the radial head. The radius alignment was equal on both sides. A comparison of the left and right ulnae revealed a clear retrocurvated malposition in the proximal shaft area of the affected side. To correct this, an osteotomy of the ulna was performed using PSIs. The preoperative 3D planning did not aim for an equal-sided ulnar alignment but at a correctly positioned radial head. Postoperative CT showed a better position of the radial head in relation to the capitulum radii; however, the full planning goal was not achieved (Figure 4)

After the operation, the patient was pain-free without instability, and the ‘click’ during ROM examination had disappeared.

### Case 5: Forearm deformity with radial head dislocation in multiple hereditary exostoses

The 8-year-old boy presented at our hospital with pain and an extension deficit of 30° in the left elbow, as well as reduced supination and bowing of the left forearm. The patient had multiple hereditary exostoses.

**Figure 5:**
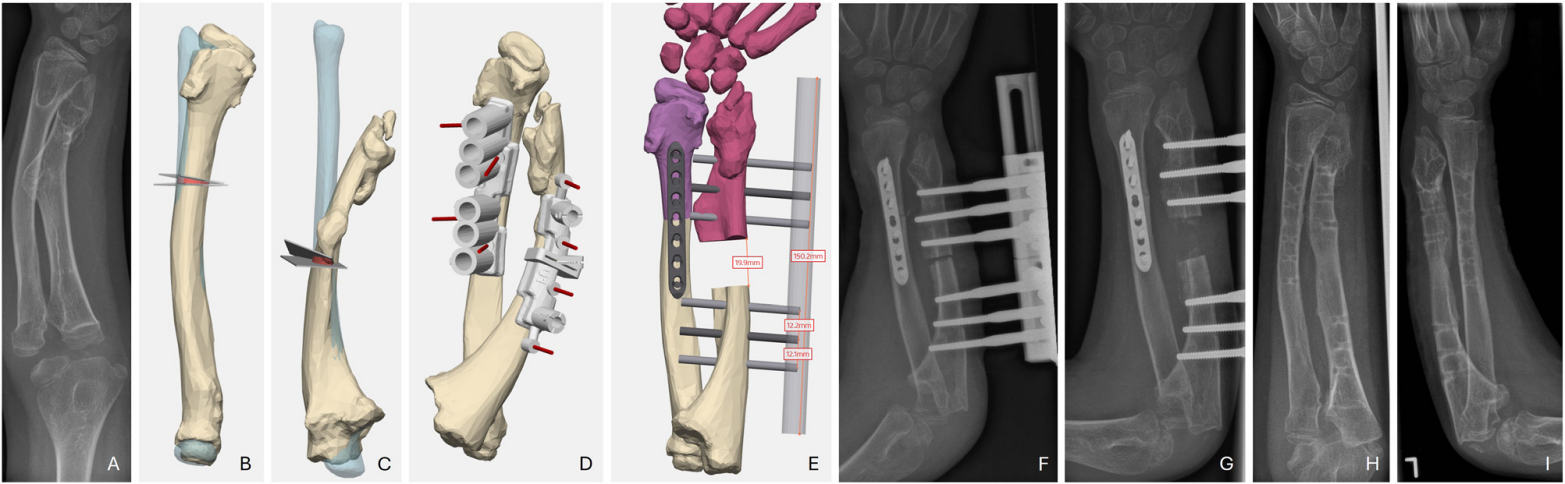
Case 5. A preoperative anterior-posterior radiograph; B planning radius osteotomy based on the age-appropriate reference model; C planning ulnar osteotomy based on the age-appropriate reference model; D PSIs; E outcome simulation; F postoperative radiograph before lengthening; G radiograph at the end of ulnar lengthening; H and I radiographs after removal of plate and fixator.

Radiography of the left forearm revealed a situation similar to that of type IIb according to Masada [17]. The deviation of the forearm bones from the age-appropriate reference model is shown in Figure 5. During corrective osteotomy, exostoses on the ulnar shaft and distal radius were removed. The placement of the bone screws for the external fixator for ulnar lengthening and ulnar osteotomy was performed using a drill and saw guide. During 3D planning, it became apparent that the ulnar osteotomy also required ulnar rotational correction, which would have been very difficult to achieve without guidance. Radius correction was also performed using PSIs. There were no problems during ulna lengthening. The plate and fixator were removed seven months later (Figure 5)

The patient subjectively profited from the surgery with a reduction in pain and a straight forearm axis, although the ROM only improved slightly, reaching Ex/Flex 0/20/140. Pro- and supination were each possible up to 10° with a primarily neutral forearm position. The radius head repositioning did not occur to the desired extent. It is possible that insufficient attention was paid to the reduction of the radial head during planning. The planning was based solely on age-appropriate reference models without a simulation of radial head repositioning.

A tabular summary of all cases can be found in Supplementary Table 1.

## Results of narrative literature review

The literature search with the strategy listed in Table 1 yielded 208 hits. After a title/abstract screening, 60 articles were selected for full-text screening.

**Figure 6:**
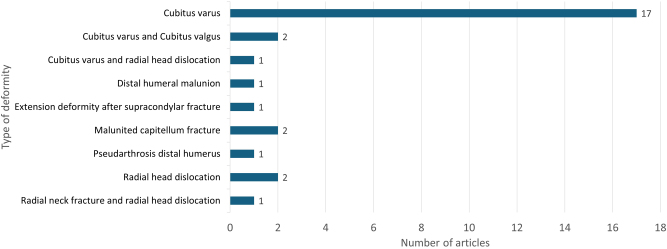
Frequency of articles per deformity.

After full-text screening, 28 studies presenting results with 3D-based surgical techniques for corrective osteotomies of the elbow region, including the use of PSIs, were filtered out. The correction of cubitus varus deformity was described in 17 articles, making it the most frequently described deformity. Figure 6 shows the corrected elbow deformities. The ages of the patients for whom the methods were used during treatment ranged from 3 [18], [19], [20] to 59 [21] years. The longest follow-up period described was 4.8 years [19]. Four comparative studies [20], [22], [23], [24], 15 case series (number of included patients: 2–30) [6], 19], 21], [25], [26], [27], [28], [29], [30], [31], [32], [33], [34], [35], [36] and 9 case reports [18], [37], [38], [39], [40], [41], [42], [43], [44] were found (Supplementary Table 2).

*Cubitus varus deformity:* Four comparative studies were identified [20], [22], [23], [24]. These studies compared the correction of cubitus varus using conventional surgical techniques with 3D-based techniques. In terms of correction accuracy, the 3D-based surgical method showed significantly better results in the studies of Hu et al. (35 cases) [22], Li et al. (40 cases) [20] and Zou et al. (32 cases) [24]. However, Zhang et al. (25 cases) found no significant difference in the postoperative carrying angle between the two groups [23]. Hu et al. and Li et al. found no clinical differences between the two groups Zhang et al. and Zou et al. did. Significantly shorter operating times were described by Hu et al. [22], Zhang et al. [23] and Zou et al. [24]. Furthermore, Zou et al. [24] reported that less radiation exposure was achieved in the navigation template group. Larger case series with 15 or more cases have been published for the correction of cubitus varus deformity by Omori et al. (17 cases) [25], Takeyasu et al. (30 cases) [26] and Yan et al. (15 cases) [19]. Low error rates in accuracy and good to excellent clinical results have been reported.

*Radial head dislocation/neglected Monteggia fracture/malunion of the radial neck:* Mueller et al. (one case) [37], Oka et al. (two cases) [27] and Weigelt et al. (three cases) described how 3D techniques can be successfully performed in individual cases of chronic dislocation of the radial head.

*Malunited intra-articular fractures:* Kholinne et al. (one case) [38] and Oura et al. (two cases) [21] described the use of 3D techniques for intra-articular elbow malalignment with good clinical and radiological outcomes.

*Malunited extra-articular fractures:* The review by Michielsen et al. included one case [39] describing the correction of pseudarthrosis of the distal humerus with severe malalignment, and Oura et al. (three cases) [28] used 3D methods to correct hyperextension malalignment after a supracondylar humerus fracture. The correction of CVL deformity was mentioned in studies by Tricot et al. (one case) [29] and Xue et al. (seven cases) [30] with successful implementation.

## Discussion

This case series with a review of the literature demonstrates the possible applications of 3D-based planning and surgical methods for bony deformities of the elbow joint. Our case series presents the correction of the following deformities: cubitus varus, minimal dorsal radial head subluxation, translational deformity of the distal humerus, extreme CVL and radial head dislocation in HME. The Supplementary literature presents further deformities that were corrected on a 3D basis: intra-articular fracture of the capitulum radii, extension deformity of the distal humerus and malunited radial neck fracture.

Extra-articular corrective osteotomies of the distal humerus, especially those for cubitus varus using 3D-based methods and PSIs, showed satisfactory clinical and radiological results in our case studies and in the literature. Comparative studies have confirmed higher correction accuracy for 3D planning [20], 24]. It is highly likely that higher correction accuracies correlate with clinical improvements, which was demonstrated for the correction of paediatric malunited both-bone forearm fractures by Roth et al. [45]. Accordingly, in a systematic review by Hoffman et al. on surgical techniques for cubitus varus correction in children, it was found that the 3D-supported technique was the only one that addressed deformity in the sagittal, coronal and transverse planes; 87.8 % of the patients treated with this technique showed excellent results, with only 2.2 % showing poor results. Compared to the other techniques analysed (lateral wedge osteotomy, step-cut osteotomy, dome osteotomy and distraction osteogenesis), this technique had the lowest percentage of poor results [46]. Nevertheless, two of the four comparative studies in the literature review show no significant differences in elbow function [20], 22], and one shows none in the radiological [23] results with regard to the arm axis.

However, in our opinion, complex extra-articular distal humeral deformities, such as in Cases 1–3, are very difficult to correct with anatomical precision using conventional methods. The extra-articular translational deformity of the distal humerus treated in Case 2 of our case series is an excellent indication for surgery with PSIs. The planning of such precise saw guidance would not have been possible using conventional planning and execution, and the patient would probably not have received a cosmetically acceptable outcome. In this case, the fact that the existing screw holes could be used intraoperatively as a reference for guide placement also simplified the procedure.

Although the deformity was present both intra- and extra-articularly in Case 3, we decided to correct the extra-articular component alone, as the joint mobility was almost unrestricted and neurological and cosmetic problems were the focus for the patient. In this case, visualisation of the deformity and the possibility of simulating several approaches for correction were very helpful. An implementation without PSIs was favoured here to have room for manoeuvres in the operating theatre with regard to the correction of the arm axis, depending on how the tension of the soft tissue develops during correction.

While corrective osteotomy of the cubitus varus and other extra-articular deformities using 3D techniques are often described in the literature and have already been well studied, we would also like to draw attention to the possibility of correcting other elbow deformities, particularly radial head dislocation. Two of our cases (1 and 4) showed minimal dorsal subluxation of the radial head, which clinically led to discomfort and a feeling of instability. Patient four had already undergone arthroscopy without recognising radial head subluxation as a possible cause. Only the 3D reconstruction, particularly the comparison with the contralateral side, revealed minimal deviations in the humeroradial joint and impressive deviations in the ulnar alignment. Both cases showed ulnar recurvation compared to the opposite side. Approximately 25–50 % of Monteggia fractures in children are unrecognised [47]. Initially, persistent dislocation of the radial head is not very symptomatic. However, in the long term, pain, instability and restricted ROM manifest themselves [48]. Based on this fact and the two cases described, it is worthwhile, from today’s perspective, to carry out 3D analyses of elbow pain in adulthood after childhood forearm fractures if no other obvious cause of the complaint has been found. To our knowledge, there are no studies with long-term follow-up of paediatric forearm fractures to adulthood or, conversely, studies on adults with elbow pain who have had a forearm fracture as a child.

The great advantage of the 3D analysis method in a side-by-side comparison or based on age-appropriate templates is that the exact extent of the deformity can be reliably recognised. The implementation of surgical correction using PSIs is not necessarily 100 % accurate [31], 49], 50]. This is also evident in Case 4. With such minimal deviations and osteotomy wedges as in Case 4, the slightest deviations in the positioning of the guides during the operation may be decisive, and exact intraoperative guide placement on the real bone without minimal displacement is not always easy. While Case 1 could be reconstructed exactly, Case 4 was slightly under-corrected with regard to the repositioning of the radial head. In addition to the exact guide placement, it is highly conceivable that soft tissue or additional cartilaginous structures, which are not considered in planning with CT, also influence the real correction accuracy. However, there are many views on how to approach chronic Monteggia lesions, and incomplete centring of the radial head after surgical reconstruction attempts must be expected [51]. Furthermore, in 3D planning for radial head dislocations, the extent to which planning should be carried out on the age-appropriate template/the opposite side or 3D planning in such a way that the radial head is securely centred in the computer simulation must be discussed. The latter seems more logical at first, but in Case 4, it did not lead to the desired result. A combination of conventional surgical techniques, in which success rates of >80 % are described [52], and 3D methods may be a good approach – planning the ulnar osteotomy height and the wedge using PSIs, then setting the radial head under the capitulum using an open approach, and only then performing osteosynthesis of the ulna with the planned plate without having pre-drilled the screws.

In Case 5, radial head relocation in the Masada type II situation in HME was not achieved with our procedure. The problem of persistent radial head dislocation in these patients after ulnar lengthening has also been described by other authors [53], [54], [55], [56]. Cao et al. [56] reported that with their method, no redislocations of the radial head were observed in all 20 patients with Masada type II forearm deformity after a mean follow-up of 36 months. However, the reduction of the radial head was equated with a position at the level of the ulnar coronoid. To what extent this means a safe humeroradial articulation cannot be assumed from our point of view after prolonged radial head dislocation with deformity and soft tissue interposition. Cao et al. [56] suggested that once the radial head dislocates, surgical attempts for relocation should be performed quickly. Our patient had a radial head dislocation for some time. Cao et al. [56] used an Ilizarov ring fixator instead of a unilateral fixator on the ulnar bone. Initially, a transfixing wire is inserted over the distal radioulnar joint during lengthening, which is removed once the radial head reaches the level of the ulnar coronoid process. A proximal ulnoradial wire was placed for further ulnar lengthening after removing the initial distal transfixing wire. The combination of the procedure of Cao et al. [56] with a 3D-supported procedure could optimise the results – a possibly necessary ulnarotational and radial osteotomy, as well as the necessary steps for better radial head centring, could be simulated.

**Figure 7:**
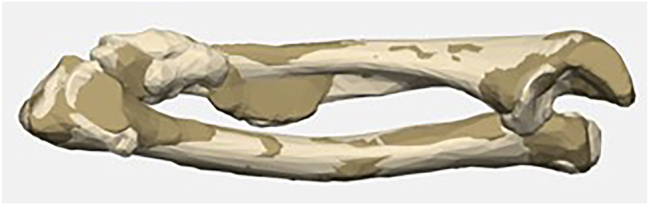
Case 5: Comparison of CT-generated model (light) and MRI-based model (dark).

In this context, one disadvantage of the 3D planning method we have used so far is that cartilaginous structures (i.e. also the cartilage cap of the exostoses) are not captured to full extent in the CT-supported segmentation. In their paediatric cubitus varus case, Bovid et al. [18] described the segmentation and generation of bone models using magnetic resonance imaging (MRI). This seems promising for the treatment of HME-related deformities of the forearm in the future, as it could also be used to consider cartilaginous structures. We also compared the segmentation results of CT and MRI in Case 5 (Figure 7). As expected, MRI-based segmentation did not fully correspond to CT-based segmentation. However, the planning and performance of paediatric osteotomies based on MRI have not yet been sufficiently validated and approved by the providers we use. However, significant development potential is expected in the future.

The successful corrections of malunited capitulum fractures described in the literature appear plausible, although only three cases were described in the two studies. Similar to other authors, we have successfully applied this method for intra-articular malunion of the wrist [57], 58].

The following general pitfalls regarding the performance of corrective osteotomies with PSIs must be summarised [1] Careful examination of the planned guide positioning is mandatory, since suboptimal intraoperative guide positioning is most likely the main cause of incomplete correction [49]. [2] Planning is carried out solely on the bone. The role of soft tissue, cartilage and, in particular, the ligament structures of the humeroradial joint is not taken into account. [3] Some surgery plans contain pre-bent plates. Pre-bending is carried out using the outcome models provided. The slightest deviations in pre-bending also lead to correction deviations [31]. [4] The use of pre-drilled screw holes do not guarantee adequate screw positioning [49]. [5] When planning joint malalignments, as is the case with dislocations of the radial head, it must be considered in advance whether the respective bones of the opposite limb or the reduced joint position should serve as a reference. A final recommendation on this matter cannot yet be given.

The limitation of our case series with a literature review is that prior to the study objective, the presentation of current and prospective surgical options with PSIs at the elbow, only example cases were described. This was not a retrospective or prospective observational study. This is a narrative review, not a quantitative data synthesis. Only the PubMed database was used for the review, which does not comply with the PRISMA guidelines and therefore does not constitute a systematic review. However, the most important publications on this topic should still be included. A structured systematic review with quality assessment would not have been feasible given the small number of publications, which are mainly case reports and case series, and would not have been in line with the general objective of summarising the current state of published research. Furthermore, it can be assumed that only good results will be published, not failures and poor results. However, the good results shown should be sufficient to further develop the method and investigate it in larger retrospective or prospective studies. It is important to define clear outcome criteria at several levels for this: clinical with elbow scores, radiological including 3D accuracy and patient-reported outcome measurements. In the present case reports, no standardised results with PROMs could be presented, as these were not routinely collected and documented in the medical records in everyday clinical practice. Nevertheless, the positive and negative subjective and objective outcome data were described comprehensively in the case reports. Long-term results must be considered when calculating how much more expensive the use of the method is, for example, if the costs of osteoarthritis treatment are avoided. Furthermore, the use of artificial intelligence has been a focus of computer-assisted surgery, leading to greater automation and possibly time and cost reduction in surgical planning [59]. The aim of the study was to present the current state of knowledge from the literature and our own experience regarding the use of PSIs in the elbow region, particularly in corrective osteotomies. Using our own illustrative case series, we highlight the potential possibilities of the procedure without making generalisations that are only possible based on larger studies.

## Conclusions

The application of 3D-based planning methods, including the use of PSIs, offers excellent results for corrective osteotomies of the elbow joint. Superiority to previous treatment methods was demonstrated by the majority of studies found in our literature search. Beyond the correction of cubitus varus, the method is currently used primarily for severe deformities, although only individual case reports are available. In particular, the extension of 3D planning for the correction of chronic dislocation of the radial head after Monteggia fractures and in HME appears promising. In the case of elbow complaints in adulthood and a history of trauma to the forearm in childhood, 3D analysis and a side-by-side comparison of the two arms are worthwhile. Previously conservatively treated forearm fractures may conceal Monteggia-like fractures that have been overlooked in conventional radiography because of minimal subluxation of the radial head. Further development of MRI-based planning is highly desirable. Artificial intelligence will also play an important role in the investigation and application of this method in the future, particularly to speed up and simplify the planning process.

## Supplementary Material

Supplementary Material
