# Supplementary material for: Strategies for surgical reconstruction of complex elbow deformities based on patient-specific instruments – a case series and narrative review
Source: Innov Surg Sci. 2025 Nov 17;11(1):15–26. doi: 10.1515/iss-2025-0024 (PMC12908972; doi:10.1515/iss-2025-0024)
Supplement: Supplementary file 1 — Supplementary Material [file j_iss-2025-0024_suppl_001.pdf]

Strategies for surgical reconstruction of complex elbow deformities based on patient-specific instruments – a case series and narrative review

Sabine M.I. Roth, Christian D Weber, Frank Hildebrand and Heide Delbrück

Supplementary material

Supplementary Table 1: Summary of the five cases.

Case	Age	Gender	Deformity	Deformity origin	Complaints	Therapy	Outcome
1	38	m	cubitus varus, dorsal subluxation of the radial head	fracture as a child	elbow pain	corrective osteotomy of the distal humerus and the proximal ulna	no pain, free ROM, exact match of 3D planning und postoperative CT
2	27	f	translational malalignment of the distal humerus	supracondylar humerus fracture as a child	elbow pain, inability to lift loads	corrective osteotomy of the distal humerus	no pain, ability to lift loads, straight arm, good ROM
3	33	f	cubitus valgus 60°	elbow fracture in early childhood	numbness, loss of strength, arm misalignment (stretching of the ulnar nerve)	extensive corrective osteotomy of the distal humerus	no further neurology, unrestricted ROM, straight arm
4	18	f	dorsal subluxation of the radial head	forearm fracture as a child	elbow pain	corrective osteotomy of the proximal ulna	no further pain, no complete correction in comparison of 3D planning and postoperative CT
5	8	m	forearm type IIb according to Masada	multiple hereditary exostoses	elbow pain, extension deficit, reduced supination	corrective osteotomy of the radial shaft, lengthening osteotomy of the ulna shaft with correction of bowing and rotation	pain reduction, straight forearm axis, only slight improvement of ROM, no full radius head reposition

Supplementary Table 2: Studies reporting results of 3D-based surgical techniques for corrective osteotomies in the elbow region

<table>
<tr><th>Author, publication year</th><th>Article type</th><th>Number of presented cases[b)]</th><th>Type of deformity;</th><th>mean age (years); range</th><th>mean follow-up (years); range or ±SD</th></tr>
<tr><td>Alonso et al., 2024(50)</td><td>case series, technical note</td><td>3</td><td>CVR</td><td>10.0; 9.0-12.0</td><td>0.5; 0.3-0.9</td></tr>
<tr><td colspan="6">Main statement: 3D computer modelling with the development of customised drilling and cutting guides allows complex medial opening wedge osteotomy for the correction of cubitus varus deformity in immature children with best-fit plate synthesis.</td></tr>
<tr><td>Barbier et al., 2019(51)</td><td>case series</td><td>5</td><td>CVR</td><td>10.2; 4.0-15.0</td><td>0.5; ns</td></tr>
<tr><td colspan="6">Results: Cubitus varus rectification was satisfactory in all five patients at 6 months postoperatively. Comparison at 6 months postoperatively between planned and measured carrying angles showed errors of 0°, 4°, 1°, 1°, and 7°, respectively.</td></tr>
<tr><td>Bovid et al., 2019(18)</td><td>case report</td><td>1</td><td>CVR</td><td>3.0</td><td>2.3</td></tr>
<tr><td colspan="6">Results: symmetric range of motion, radiographs confirmed appropriate alignment and healing; participating in all activities as desired and denied pain</td></tr>
<tr><td>Delbrück et al., 2022(38)</td><td>case series</td><td>3[b)]</td><td>CVR, radial head dislocation (RHD)</td><td>23.3; 15.0-37.0</td><td>1.3; 1.2-1.5</td></tr>
<tr><td colspan="6">Results: good patient satisfaction, improvement of DASH and 3D-radiological results in part comparison analysis</td></tr>
<tr><td>Gemalmaz et al., 2017(52)</td><td>case report</td><td>1</td><td>CVR</td><td>18.0</td><td>0.3</td></tr>
<tr><td colspan="6">Results: full ROM, no pain, osteotomy union</td></tr>
<tr><td>Hu et al., 2020(22)</td><td>comparative study</td><td>35 (16 3D-supported; 19 conventional (CV))</td><td>CVR</td><td>7.5; 4.6–13.2</td><td>0.5-1.0</td></tr>
<tr><td colspan="6">Results:
• average operation times: 11.7 min (9.6–13.5 min) 3D-group and 22.89 min (17.7–26.8 min) CV-group (p < 0.001).
• average differences in postoperative carrying angles between affected and healthy sides: 1.13° (0–2.0°) 3D-group and 4.21° (0–7.5°) CV group (p < 0.001).
• elbow function did not differ significantly between groups using the Bellemore criteria (p > 0.05)</td></tr>
<tr><td>Kholinne et al., 2024(29)</td><td>case report</td><td>1</td><td>malunited capitellum fracture</td><td>54</td><td>1.0</td></tr>
<tr><td colspan="6">Results: patient high satisfaction</td></tr>
<tr><td>Li et al., 2022(20)</td><td>comparative study</td><td>40 (20 3D-supported; 20 CV</td><td>CVR</td><td>CV: 9.3±6.2
3D: 8.9±3.3</td><td>CV: 3.1±1.0
3D: 3.1±0.9</td></tr>
<tr><td colspan="6">Results: Closing-wedge osteotomy conventional vs. 3D-printed model and osteotomy template: The 3D-printed template technique showed better osteotomy accuracy, but no significant advantage in terms of functional and cosmetic results than conventional osteotomy.</td></tr>
<tr><td>Marinelli et al., 2022(53)</td><td>case series</td><td>2</td><td>CVR</td><td>27.0 (16.0-38.0)</td><td>1.8; 1.5-2.0</td></tr>
<tr><td colspan="6">Main statement: The precision of computer-aided surgical planning and custom-made surgical guides allows for reproducible and relatively safe surgeries, even in extreme deformities, where surgical complexity could discourage attempts at surgical correction.</td></tr>
<tr><td>Michielsen et al., 2019 (30)</td><td>case report[b)], review</td><td>1[b)]</td><td>pseudarthrosis distal humerus with severe deformity</td><td>22.0</td><td>2.0</td></tr>
<tr><td colspan="6">Results: good alignment, VAS 2, continued physiotherapy was necessary to regain a functional arc of motion</td></tr>
</table>

Mueller et al., 2017 (27)	case report	1	malunited ulna fracture and RHD after Monteggia fracture six months ago	23.0	ns
**Main statement:** PSIs as a navigational aid for reconstructive surgery of ulnar fracture combined with allograft surgery of the radial head and radial condyle yielded satisfactory results.					
Oka et al., 2012(28)	case series	2	chronic RHD with malunited fractures of both bones	14.5; 14.0-15.0	4.0; 3.5-4.5
**Results:** normal range of forearm rotation with almost completely reduced position of the radial head					
Oka et al., 2019(6)	case series	5[b)]	distal humeral malunion	22.2;12.0-47.0	1.0
**Results:** Mean deformity angle preoperative 29.8 ± 3.5 vs. postoperative 3.0 ± 1.3; 3D corrective osteotomy using PMI resolved treatment challenges for complex deformities in the upper extremities.					
Oka et al., 2024(54)	review + case presentation	2	CVR	12.0; 9.0-15.0	ns
Main statement: 3D computer simulations and the use of patient-matched instruments for cubitus varus deformity are reliable and can facilitate accurate and safe correction.					
Omori et al., 2015 (25)	case series	17	CVR	17.6; 8.0-45.0	2.0; 0.7-3.6
**Results:** Mean errors in 3D corrective osteotomy were 0.6°±0.7° in varus-valgus rotation, 0.8°±1.3° in flexion-extension rotation, 2.9°±2.8° in internal-external rotation, 1.7±1.8 mm in anterior-posterior translation, 1.3±1.8 mm in lateral-medial translation, and 7.1±6.3 mm in proximal-distal translation. The 3D correction of cubitus varus deformity was performed accurately within the allowable error limit.					
Oura et al., 2016 (21)	case series	2	malunited intra-articular fracture of the distal humerus (articular step-off in the capitulum)	45.0;31.0-59.0	2.0
**Results:** No complaints of pain or instability were observed. ROM 5°–140° and 15°–125°, respectively. Plain radiography and CT revealed good reconstruction of the articular surface.					
Oura et al., 2018 (31)	case series	3	extension deformity of the distal humerus after a malunited supracondylar fracture	26.3;12.0-55.0	3.4;2.6-4.1
**Results:** The range of flexion/extension of the elbow motion in the first patient improved from 95°/25° preoperatively to 140°/–10° postoperatively, in the second patient from 100°/20° preoperatively to 145°/5° postoperatively, and in the third patient from 80°/25° preoperatively to 140°/10° postoperatively.					
Sedigh et al., 2021 (55)	case report	1	CVR: The wedge was flipped 180° in the coronal plane. The medial border of the wedge was aligned with the lateral border of the humeral shaft.	18.0	0.3
**Results:** The clinical carrying angle was 5° valgus. The patient was completely satisfied with the surgical outcome.					
Sri-Utenchai et al., 2021(56)	case report	1	CVR	32	1.5
**Results:** Postoperative radiographs demonstrated an improved humerus-elbow-wrist angle, from 15º varus to 7º valgus. The patient was highly satisfied.					
Takeyasu et al., 2013(26)	case series	30	CVR	15.1; 4.0-49.0	1.9; 1.0-4.4
**Results:** The mean humerus-elbow-wrist angle and tilting angle on the affected side improved significantly from 18.2° (varus) and 25.0° before surgery to 5.8° (valgus) and 38.0° after surgery. Hyperextension of the elbow and internal rotation of the shoulder were normalised in all the patients. Twenty-seven patients had excellent results, three had good results, and none had poor results.					

Tricot et al., 2012(32)	case series	3	2 CVR, 1 CVL	10.3; 8.0-13.0	0.5
**Results:** All patients were asymptomatic and regained full elbow mobility. Satisfactory corrections were obtained for all cases.					
Wan et al., 2021 (57)	case report	1	CVR	12.0	0.8
**Results:** The accuracy of the osteotomy angle was confirmed using postoperative radiography.					
Weigelt et al., 2017 (58)	case series	4	malunited RNF with/without RHD	12.0; 10.0-16.0	1.3; 1.0-2.0
**Results:** Deformity correction of 58 %–89% was achieved; despite the lack of improved ROM, all patients were satisfied. Preoperative subluxation of the radial head was corrected in two of three patients. No signs of avascular necrosis of the radial head were observed in any patient.					
Xue et al., 2023(33)	case series	17 patients, 18 elbows	7 CVL, 11 CVR; planning on 3D printed individual models, no PSIs	26.4; 18.0-44.0	1.2; 1.0-1.6
**Results:** All patients underwent the operation successfully and had no postoperative deformity. The function of the elbow joint was excellent in seven cases and good in ten cases.					
Yan et al., 2024 (19)	case series	15	CVR	9.7; 3.0-17.0	3.3; 2.0-4.8
**Results:** The mean preoperative humeral-elbow-wrist (HEW) angle of the affected side was -21.7° (ranging from -14° to -34°), while that of the normal side was 9.4° (ranging from 5° to 15°). The postoperative HEW on the affected side was 9° (ranging from 4° to 16°). There was no significant difference between the normal and affected sides after surgery (p = 0.74). Fourteen patients (93.3%) were satisfied with the overall appearance of their elbow. None lazy-S deformity was observed in these cases.					
Zhang et al., 2011(59)	case series	18	CVR	15.7; 13.0-19.0	1.5; 1.0-2.0
**Results:** A total of 15 patients (83.33%) were very satisfied with the outcome, 2 (11.11%) were satisfied, and 1 (5.56%) was dissatisfied with the outcome.					
Zhang et al., 2019 (23)	comparative study	25 (14 3D-supported; 11 CV	CVR	CV: 9.6; 6.0–17.0<br>3D: 9.9; 5.0–18.0	CV: 1.5±0.3<br>3D: 1.5±0.2
**Results:** Compared with the conventional group, the 3D printing group had the advantages of shorter operation time, less intraoperative blood loss, higher rate of excellent correction, and higher rate of the excellent satisfaction with appearance after deformity correction (p < 0.001, p < 0.001, p = 0.019, p = 0.023). Nevertheless, no significant difference was observed in the postoperative carrying angle of the deformed side or the total complication rate between the two groups (P = 0.626, P = 0.371).					
Zou et al., 2024 (24)	comparative study	32 (17 3D-supported; 15 CV	CVR	9.5; 8.0-14.0	ns
**Results:** More accurate osteotomy degrees, shorter operation time, and less radiation exposure were achieved in the navigation template group (p < 0.05). At the last follow-up, a significant difference was found based on the Bellemore criteria(59, 60) (p = 0.0288).					

[a)]regarding elbow and forearm; [b)]only elbow cases considered; CV conventional; DASH Disabilities of Arm, Shoulder and Hand; VAS Visual Analogue Scale; CVR cubitus varus; CVL cubitus valgus; RHD radial head dislocation; RNF radial neck fracture
